# Lipid Coated Microbubbles and Low Intensity Pulsed Ultrasound Enhance Chondrogenesis of Human Mesenchymal Stem Cells in 3D Printed Scaffolds

**DOI:** 10.1038/srep37728

**Published:** 2016-11-24

**Authors:** Mitra Aliabouzar, Lijie Grace Zhang, Kausik Sarkar

**Affiliations:** 1 Department of Mechanical and Aerospace Engineering, The George Washington University, Washington, DC, 20052, USA; 2 Department of Biomedical Engineering, The George Washington University, Washington, DC, 20052, USA; 3 Department of Medicine, The George Washington University, Washington, DC, 20052, USA

## Abstract

Lipid-coated microbubbles are used to enhance ultrasound imaging and drug delivery. Here we apply these microbubbles along with low intensity pulsed ultrasound (LIPUS) for the first time to enhance proliferation and chondrogenic differentiation of human mesenchymal stem cells (hMSCs) in a 3D printed poly-(ethylene glycol)-diacrylate (PEG-DA) hydrogel scaffold. The hMSC proliferation increased up to 40% after 5 days of culture in the presence of 0.5% (v/v) microbubbles and LIPUS in contrast to 18% with LIPUS alone. We systematically varied the acoustic excitation parameters—excitation intensity, frequency and duty cycle—to find 30 mW/cm^2^, 1.5 MHz and 20% duty cycle to be optimal for hMSC proliferation. A 3-week chondrogenic differentiation results demonstrated that combining LIPUS with microbubbles enhanced glycosaminoglycan (GAG) production by 17% (5% with LIPUS alone), and type II collagen production by 78% (44% by LIPUS alone). Therefore, integrating LIPUS and microbubbles appears to be a promising strategy for enhanced hMSC growth and chondrogenic differentiation, which are critical components for cartilage regeneration. The results offer possibilities of novel applications of microbubbles, already clinically approved for contrast enhanced ultrasound imaging, in tissue engineering.

Cartilage regeneration remains one of the prized goals of tissue engineering because of the notorious difficulties of the cartilage repair process^1^. In recent years, tissue engineering approaches have focused on human mesenchymal stem cells (hMSCs) as an important cell source^2^,^3^,^4^,^5^ for their ability to differentiate into many cell types^6^. The underlying guiding principle is that the hMSCs would differentiate into a particular cell type and grow the corresponding tissue when directed by the appropriate chemical (e.g. appropriate growth factors) and mechanical cues supplied in the environment^7^,^8^,^9^. Cartilage and bone cells are known to respond favorably to mechanical stresses due to ultrasound^10^,^11^,^12^,^13^. Here we have investigated for the first time the effects of lipid-coated microbubbles (MBs) in presence of low intensity pulsed ultrasound (LIPUS) on chondrogenic differentiation of hMSCs in a three-dimensional (3D) printed scaffold.

Over the past few decades, the medical use of ultrasound has been extended beyond imaging and diagnosis towards therapeutic applications. Therapeutic effects of LIPUS, with frequency ranging from 0.75 to 1.5 MHz and intensity lower than 100 mW/cm^2^, have been investigated in a number of applications including fracture healing^10^,^14^,^15^,^16^, wound healing^17^ and treatment of glaucoma^18^. Although LIPUS has been approved for treatment of fresh as well as non-union fractures by the US Food and Drug Administration, its practical use for cartilage repair in a clinical setting has so far been limited^19^. To shed light on this matter, several studies have investigated the effects of LIPUS on chondrocyte viability and proliferation^13^,^20^,^21^, gene expression^22^, type II collagen^23^,^24^, type X collagen^20^ and glycosaminoglycan (GAG) syntheses^25^,^26^. Parvizi *et al*. reported that LIPUS stimulation on rat chondrocytes improved aggrecan mRNA expression and proteoglycan synthesis^27^. Hasanova *et al*. demonstrated that LIPUS treated chondrocytes seeded in three-dimensional (3D) chitosan-based matrices had higher levels of type II collagen, aggrecan, L*-*Sox5 and Sox9 mRNA expression when compared to controls^28^. Increased expression of type II collagen, aggrecan mRNA, and proteoglycan synthesis, which are all crucial in regulation of chondrocytes, have been reported in past studies^23^,^24^,^25^,^29^,^30^.

Here, we have used lipid-coated MBs in order to better harness the beneficial effects of LIPUS stimulation on proliferation and chondrogenic differentiation of hMSCs. These gas-filled MBs are highly responsive to ultrasound, which has led to their application as ultrasound contrast agents (UCA)^31^,^32^,^33^. Currently, their use in facilitating delivery of therapeutics to tissues and organs through sonoporation is being actively investigated^34^,^35^,^36^,^37^,^38^,^39^. They are also being applied for embolotherapy^40^, accelerated thrombolysis^41^,^42^ and hyperthermia-induced apoptosis^43^. However, the combination of LIPUS and MBs has not been previously studied for cartilage tissue regeneration.

Most of the *in vitro* experiments investigating effects of LIPUS on cells were conducted in two-dimensional (2D) cell cultures or hydrogel thin films^12^,^44^, an environment very different from the one cells find inside the body. Recently, 3D printing has emerged as a leading technique to create tissue scaffolds incorporating intricate hierarchical structures of the native tissue as well as the patient specific geometry of target injury sites obtained by CT images^45^. Three dimensional tissue scaffolds can provide physiological environment for cells within the body unavailable in a traditional 2D setting. Here we have used a 3D tissue scaffold made of Poly-(ethylene glycol)-diacrylate (PEGDA) printed by a novel tabletop stereolithography-based technique developed in our lab^46^,^47^. PEGDA, as a UV photocurable bioink, is chosen for its high water content, biocompatibility and easy printability^48^.

The objective of the present work is to investigate the effects of LIPUS on growth and chondrogenic differentiation of hMSCs within a 3D printed matrix, in the presence of MBs. We have thoroughly evaluated the 1, 3 and 5-day proliferation as well as three-week chondrogenic differentiation of hMSCs in 3D printed scaffolds under LIPUS and MB treatment.

## Results and Discussion

### Characterization of 3D-printed scaffolds

In our previous studies without MBs, scaffolds with square channels presented highest hMSC growth and differentiation compared to other pore geometries^47^. Therefore, we chose this pore geometry for our current studies as well.

**Figure 1:**
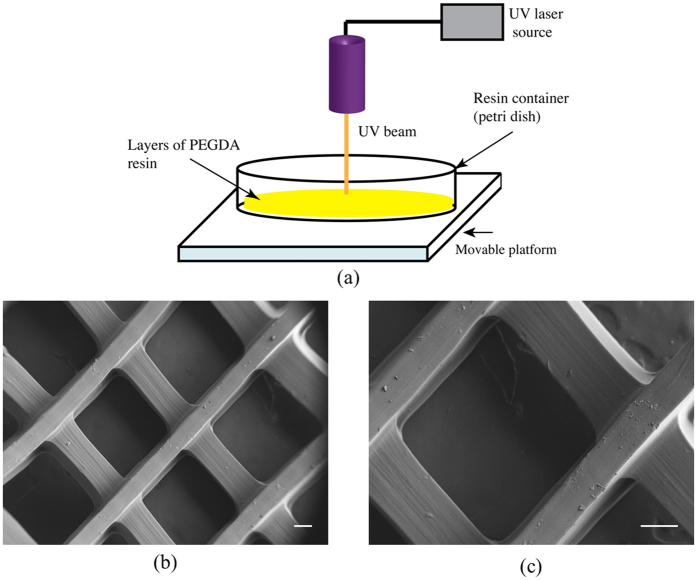
(**a**) A schematic of stereolithagraphy based 3D printer, (**b**,**c**) SEM micrographs of 3D printed cartilage scaffolds with square channels. The scale bar represents 200 μm.

Figure 1(a) shows a schematic representation of the 3D printer described in section 4.1. A scanning electron microscope (SEM, Zeiss NVision 40FIB) was employed to assess the matrix morphology and pore size. Typical SEM images of 3D-printed scaffolds with square pore shapes are presented in Fig. 1(b,c). Using Image J software (imagej.nih.gov), the pore dimension was found to be 700 × 690 μ*m*^2^. The porosity of the scaffold was 49%. We calculated the porosity by measuring its solid phase density as well as apparent mass density according to^49^. All the measurements were repeated 6 times from different positions of the printed scaffolds.

### Lipid-coated microbubbles

**Figure 2:**
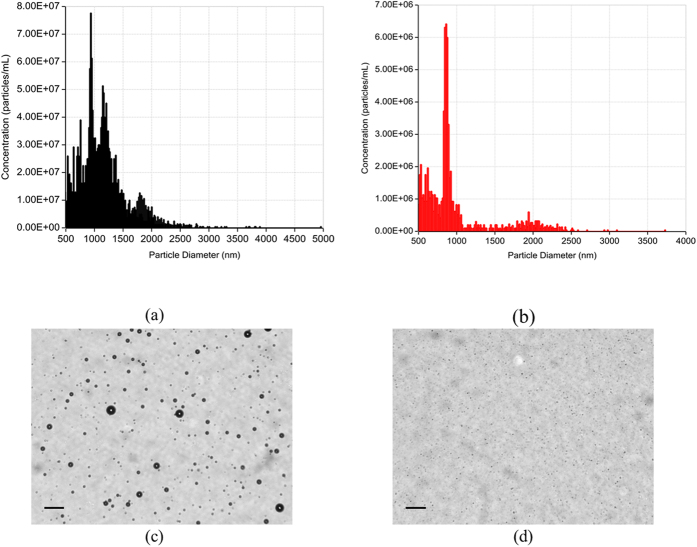
Size distribution and concentration determined using qNano system for lipid-coated microbubbles produced by mechanical agitation (**a**) before and (**b**) after centrifugation. Optical microscopy images (**c**) before and (**d**) after differential centrifugation. The scale bar is 100 *μm*.

We have used lipid-coated MBs made in-house with a perfluorobutane gas core for this study. The MB preparation method is detailed in section 4.2. Bubble production was verified by measuring acoustic scattering as described in our previous publications^33^,^50^,^51^. MBs created by the standard mechanical agitation technique have a broad size distribution as can be seen in Fig. 2(a). To make the size distribution narrower (more monodisperse), we followed a simple and rapid technique of differential centrifugation according to refs 52 and 53. Figure 2 (a,b) show that a 2-minute centrifugation at 40 relative centrifugal force (RCF) reduced the average size of MBs from 1.3 μm to 800 nm.

### Cytotoxicity of lipid-coated microbubbles

**Figure 3:**
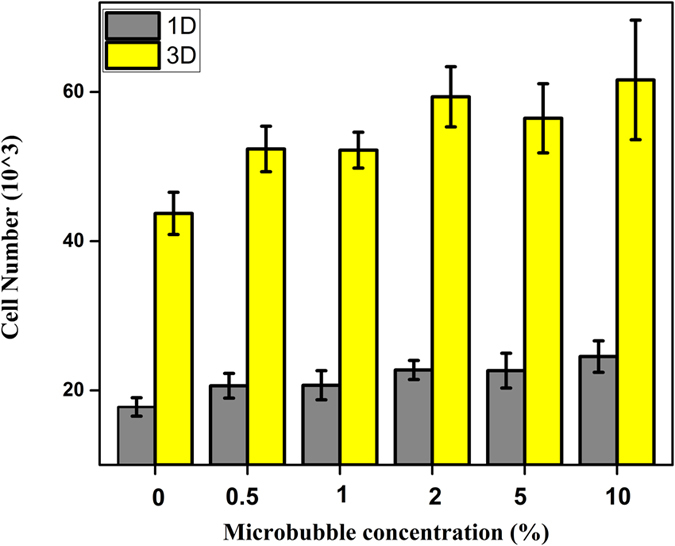
Short (1 day)- and long (3 day)-term cell viability assay results of hMSCs incubated with lipid-coated microbubbles (Data are mean ± StdEM, n = 9).

The cytotoxicity of the lipid-coated MBs synthesized in house was investigated by incubating cells with varying concentrations (0.5, 1, 2, 4, 5 and 10% v/v) of MB suspension for over 72 hours. The viability of the cells incubated with the MB suspension compared to those without MB (0% MB) was determined using the MTS assay and presented in Fig. 3. The results show an increased cell proliferation both after 24 and 72 hour time periods. Due to gas diffusion from the surrounding non-degassed PBS (phosphate buffer saline) during centrifugation, MBs were partially filled with air^54^,^55^,^56^. Observed increased cell proliferation in the presence of MBs might be ascribed to increased oxygen available to the cells. Overall, the results indicated that lipid-coated microbubbles do not cause short-term or long-term cytotoxicity to the cells at the concentrations studied here.

### Effect of LIPUS on hMSC proliferation in the presence of microbubbles

**Figure 4:**
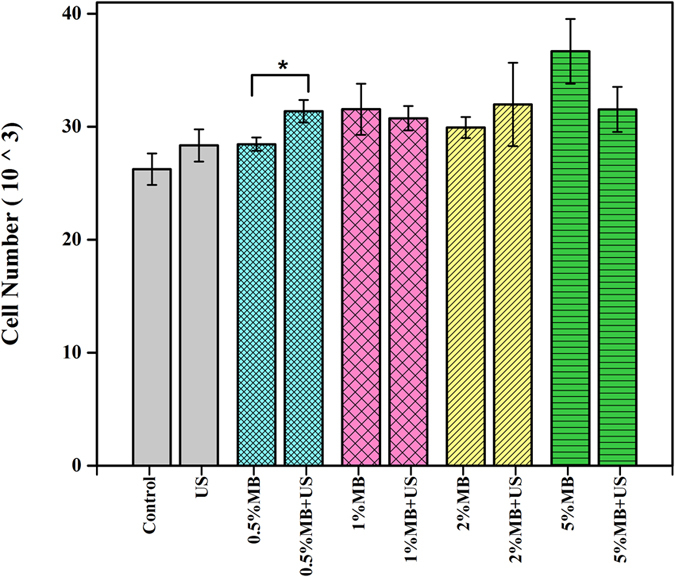
Effects of 3-min LIPUS (30 mW/cm^2^; 1.5 MHz; 20% duty cycle; 200 μs pulse length) at different concentrations of MB suspension on hMSC proliferation after 24 hrs (Data are mean ± StdEM, n = 9). Values significantly different from the control group are indicated by *for p < 0.05.

In order to determine the optimal concentration of MBs for ultrasound excitation studies, varying concentrations of MB suspension was added to the cell media. Following the addition of MBs, LIPUS (30 mW/cm^2^; 1.5 MHz; 200 μs pulse length; duty cycle 20% i.e. PRF 1 kHz) was applied for three minutes. hMSC proliferation, 24 h after LIPUS stimulation, was examined and the results are presented in Fig. 4. It shows that LIPUS alone increased cell proliferation but not significantly. However, LIPUS-treated cells in the presence of 0.5% (v/v) MB suspension resulted in the highest proliferation rate. Ultrasound in the presence of higher concentrations of MBs decreased hMSC proliferation.

**Figure 5:**
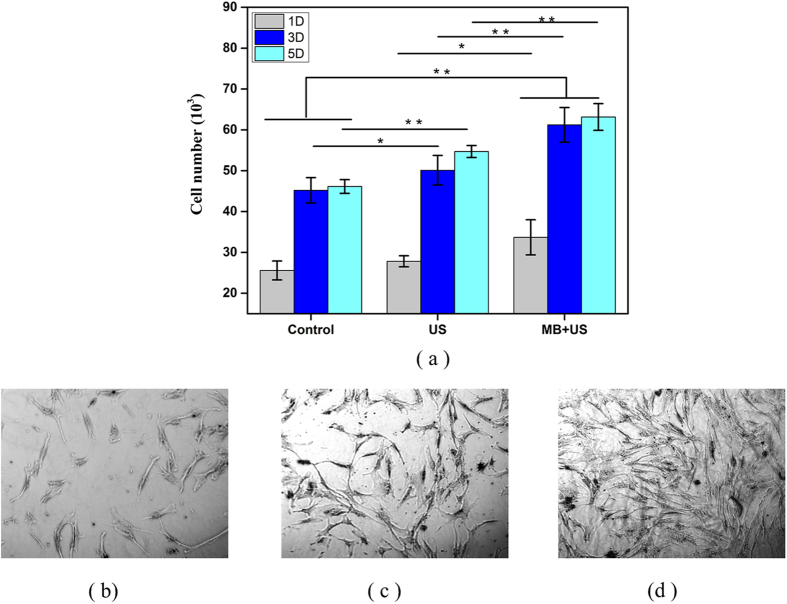
(**a**) 1, 3 and 5-day hMSC proliferation with 3-min LIPUS (30 mW/cm^2^; 1.5 MHz; 20% duty cycle; 200 μs pulse length) and 0.5% MB suspension (Data are mean ± StdEM, n = 9). Microscopic images of hMSC growth two days after LIPUS (30 mW/cm^2^; 1.5 MHz; 20% duty cycle; 200 μs) pulse length). (**b**) Control, (**c**) LIPUS, (**d**) LIPUS with 0.5% (v/v) MB suspension. Values significantly different from the control group are indicated by *for p < 0.05 and **for p < 0.01.

Consequently, we conducted 1, 3 and 5-day hMSC proliferation with LIPUS excitation in the presence of 0.5% (v/v) MB. We divided the samples into three groups: control (no LIPUS, no MB), LIPUS only, and LIPUS and MB. At predetermined time points, the cell viability was measured by an MTS assay with the results shown in Fig. 5(a). A significant increase in cell proliferation (p < 0.01) was observed with LIPUS treatment in the presence of optimal MB suspension after 1, 3 and 5 days of culture. hMSC proliferation enhanced up to 40% compared to the control (without MB and LIPUS) after 5 days of culture in the presence of MB and LIPUS while this value was only 18% when excited with LIPUS alone.

Figure 5(b), (c) and (d) show comparative microscopic images of hMSC cells—control, LIPUS without MB, and LIPUS with MB groups—demonstrating the beneficial effects of LIPUS, strongest in presence of MB.

### Determination of optimum LIPUS parameters for hMSC proliferation in the presence of microbubbles

#### Excitation intensity

**Figure 6:**
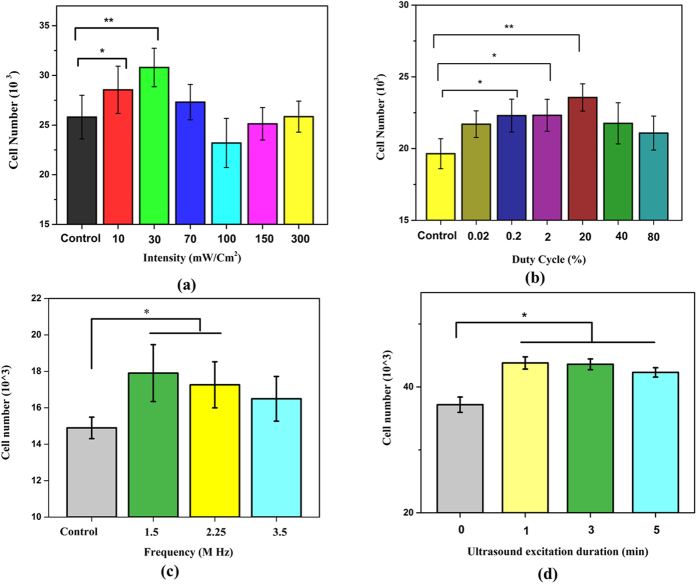
Effects of 3-min LIPUS (30 mW/cm^2^;1.5 MHz; 20% duty cycle; 200 μs pulse length unless otherwise specified for each parameter variation) stimulation at varying (**a**) intensity, (**b**) duty cycle, (**c**) frequency and (**d**) excitation duration in the presence of 0.5% (v/v) MB suspension on day 1 (Data are mean ± StdEM, n = 9). Values significantly different from control group are indicated by *for p < 0.05 and **for p < 0.01.

The optimal LIPUS excitation was determined by evaluating hMSC proliferation under various intensities (10, 30, 70, 100, 150 and 300 mW/cm^2^). Other acoustic parameters (1.5 MHz, 20% duty cycle and 200 μs pulse length) were kept the same. After 24-hour culture, hMSC proliferation was evaluated in response to 3 minutes of exposure to LIPUS in the presence of 0.5% MB suspension. As demonstrated in Fig. 6 (a), cell proliferation increased approximately by 20% at 30 mW/cm^2^ on day 1. At higher intensities, the proliferation diminished. The intensity was kept constant at 30 mW/cm^2^ for the rest of the studies. It is noteworthy to mention that our previous study showed that 100 mW/cm^2^ intensity resulted in the maximum hMSC proliferation rate without the presence of MBs (data not shown here). As expected, addition of MBs has lowered the energy threshold required for enhancing cell growth.

#### Excitation duty cycle

We investigated the effects of duty cycle over the range of 0.02–80% (i.e., pulse repetition period (PRP) over 250 μs to 1 s). The intensity, frequency and excitation duration were kept fixed at 30 mW/cm^2^, 1.5 MHz and 3 min. Duty cycle is an important, yet the least studied, acoustic parameter in the literature. It is the fraction of the time within a pulse period the transducer is transmitting (pulse length/PRP). Figure 6(b) demonstrates that at this intensity, LIPUS in the presence of MB tends to enhance hMSC proliferation when the PRP is between 1 to 100 ms, (i.e. duty cycle 20% to 0.2%). Shorter PRPs (corresponding to higher duty cycles) decreased cell proliferation. Consequently, PRP of 1 ms (duty cycle of 20%) was used for subsequent experiments.

#### Excitation frequency

To obtain the optimum excitation frequency, we varied it keeping pulse duration (200 μs) and PRP (1 ms) constant. The intensity, duty cycle and excitation period were kept fixed at 30 mW/cm^2^, 20% and 3 min. Figure 6(c) shows that all three frequencies promoted hMSC proliferation with the increase being statistically significant at 1.5 and 2.25 MHz. We chose frequency of 1.5 MHz, which has shown high promise for bone fracture healing, and kept it constant for subsequent studies.

#### Excitation duration

We also investigated the dependence of hMSC proliferation on the ultrasound excitation duration, we varied it to 1, 3 and 5 minutes following addition of 0.5% MB, keeping the intensity, duty cycle and frequency fixed at 30 mW/cm^2^, 20% and 1.5 MHz. As can be seen in Fig. 6(d) ultrasound stimulation in the presence of MB has significantly increased hMSC proliferation for all the time periods studied here compared to the control. Note that the variation in the control data across Fig. 6(a–d) are due to different donors and environmental factors.

### Effect of LIPUS treatments on hMSC chondrogenic differentiation in 3D-printed scaffolds in the presence of MB

**Figure 7:**
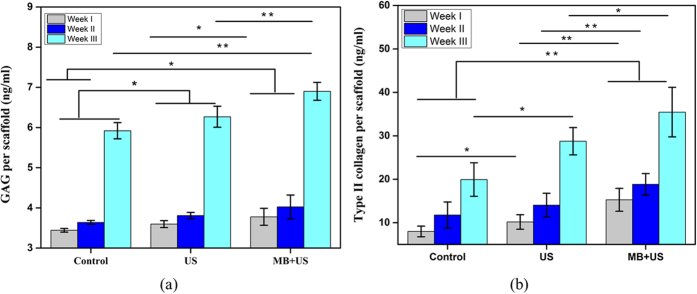
Three-week hMSC (**a**) GAG production and (**b**) type II collagen. (Data are mean ± StdEM, n = 5). Values significantly different from the control group are indicated by *for p < 0.05 and **for p < 0.01.

In our previous investigation of hMSC growth and chondrogenic differentiation without MBs, scaffolds with square pore geometry performed better than those with hexagonal pore geometry^47^. Therefore, scaffolds with square pore geometry were chosen here for hMSC chondrogenic differentiation evaluations. We divided the samples into three groups: control group (no LIPUS, no MB), LIPUS only and LIPUS + MB group. Seeded 3D-printed PEGDA scaffolds were evaluated for GAG and type II collagen after three weeks of culture. Glycosaminoglycan (GAG) and collagen type II, which are two key components of a cartilage matrix, were measured using standard assay kits following manufacturer’s instructions. Figure 7(a) shows that all LIPUS treated samples exhibited an increase in GAG production; however, the increase is significantly higher upon incorporation of MBs when compared to the controls. Samples that underwent MB assisted ultrasound excitation exhibited 17% increase in GAG production after 3 weeks. However, samples treated with LIPUS only, had a 5% increase in GAG production after 3 weeks compared to the controls.

In addition to improved GAG production, Fig. 7(b) illustrates another important cartilage matrix protein-type II collagen synthesis. Type II collagen synthesis showed 44% and 78% increase after 3 weeks for US only and US/MB groups, respectively, when compared to the controls. The results elucidate the effective role of LIPUS and MBs assisted with 3D-printed scaffolds for enhanced chondrogenic differentiation of hMSCs.

### Mechanism of cellular effects of LIPUS in the presence of MB

As we already noted, the exact mechanism behind the effects of ultrasound on cells is not completely understood. However, growing evidence suggests that mechanical stimulation in the environment of the cells significantly affects synthesis and degradation of matrix macromolecules, activates distinct regulatory pathways of metabolic functions and changes the level of transcription, translation, and post-translational modification^57^,^58^,^59^. In particular, application of fluid shear to osteoblasts—relevance of which will become clear below—induces development of thicker and more abundant actin filaments (stress fibers) as well as formation of focal adhesions containing *β*_1_-integrins and *α*-actin^60^. They in turn play a central role in signal transduction from extra cellular matrix (ECM) to the nucleus leading to an increased gene expression^61^. While dynamic compression has been shown to stimulate genes associated with chondrogenesis in hMSCs, dynamic tension was found to regulate both fibroblastic and osteogenic associated genes^62^.

Unlike steady mechanical stimulation as in tension, compression or fluid shear, LIPUS subjects the cells to a periodically varying load triggering one, many, or all of the above bioeffects. Cellular effects of LIPUS are generally categorized to be non-thermal^63^, predominantly due to cavitation, microstreaming and acoustic radiation forces^64^,^65^. Cavitation refers to the growth, oscillation, and/or collapse of gaseous cavities under excitation of acoustic waves^66^,^67^. Stable oscillations at low excitations create microstreaming, a fluid flow, around MBs causing cells to experience higher levels of shear stress, ranging between 100 Pa to 1 kPa. At higher excitations, MBs undergo nonlinear inertial cavitation, eventual bubble collapse and direct liquid jets towards nearby cell layers^35^,^38^,^68^. In fact, several *in vitro* as well as *in vivo* studies have demonstrated MBs in conjunction with ultrasound results in a process called sonoporation, transient development of pores in cell membranes^34^,^69^,^70^,^71^,^72^,^73^. The pores have been visualized by scanning electron microscopy (SEM), transmission electron microscopy (TEM) and ultrafast real time imaging^74^,^75^,^76^. In any event, we believe that, at the low excitation intensity of 30 mW/cm^2^ applied here, oscillating MBs can generate higher shear stresses on adjacent cells through microstreaming and stable cavitation. Note that ultrasound alone was also seen here to generate cellular effects though of a much lesser strength compared to when MBs were present.

## Conclusion

In this study, we designed and fabricated a novel 3D printed scaffold for cartilage regeneration. We explored for the first time the application of lipid-coated, perfluorobutane-filled microbubbles along with LIPUS as a means to enhance hMSC growth and their chondrogenic differentiation. Although microbubbles and their response in the presence of ultrasound excitation have been extensively investigated for their therapeutic effects, their applications were thus far restricted to non-tissue engineering applications. Here, it is introduced to tissue engineering, specifically for cartilage regeneration in three dimensional scaffolds. Our results show that LIPUS in combination with MB on 3D printed constructs can significantly enhance hMSC proliferation—as much as by 40%—as well as chondrogenic differentiation. Therefore, integrating LIPUS and MB appears to be a promising strategy for enhanced hMSC growth and chondrogenic differentiation.

## Methods

### Preparation of 3D-printed scaffolds

A tabletop stereolithography-based 3D bioprinter was used to fabricate structured scaffolds (Fig. 1). The printer consists of a 3D axial movable stage and a UV laser source. The printing configuration is controlled by a Pronterface control software package. It can generate different geometrical patterns using 3D computer aided design (CAD) models. Previously, we found that scaffolds with square pore patterns had higher hMSC growth and differentiation rates. Therefore, we chose the same pattern for our differentiation studies. The print speed was maintained at 25 mm/s and the laser repetition rate used to print the structured patterns varied from 8 to 11 kHz. The 3D scaffold was printed via layer by layer method. Bioink was prepared by mixing 40% (w/w) poly (ethylene glycol) (PEG, Mn 300) and 60% (w/w) poly (ethylene glycol) diacrylate (PEG-DA, Mn 575) in the presence of the photo initiator (0.5% (w/w) of PEG-DA)^77^,^78^. The morphology of the scaffolds was observed by a scanning electron microscope (SEM, Zeiss NVision 40FIB).

### Lipid-coated microbubble formulation and preparation

Lipid emulsions were formulated by dissolution of 1,2-dipalmitoyl-sn-glycero-3-phosphatidylcholine (DPPC), 1,2-dipalmitoyl-sn-glycero-3-phosphatidylethanolamine-polyethyleneglycol-2000 (DPPE-PEG-2000) and 1,2-dipalmitoyl-3-trimethylammonium propane (chloride salt; 16:0 TAP) (Avanti, AL) at a total lipid concentration of 0.75, 1.5, and 3 mg/mL. In order to have a homogenous solution of lipids, lipids were mixed in propylene glycol and heated in a sonication bath above the lipid transition temperature (45 °C) for approximately 30 minutes. PBS and glycerol were added later to the lipid solution^79^,^80^. After the lipid solution was further mixed using a magnetic stirrer, 1.5 ml of the resulting solution was added to a 3-ml glass vial. The remaining head space was gas exchanged with perfluorobutane (PFB) (Fluoromed, TX) and MBs were formed via mechanical agitation technique using Vial Mixer (Bristol Myers Squibb) for 45 seconds. After the production of MBs, we performed a two-minute centrifugation technique at 40 relative centrifugal force (RCF) to remove bubbles with larger diameters using a bucket-rotor centrifuge as described in details elsewhere^52^,^53^. The size and number of MBs were determined using the qNano (Izon Science™, MA). For the measurements, we used three nanopore membranes, NP4000: (2000 nm–10000 nm), NP2000: (1000 nm–5000 nm) and NP1000: (400 nm–1500 nm), to accurately compare the size distribution and concentration of our original and centrifuged MBs. The morphology of lipid-coated microbubbles was observed under microscope (AmScope FMA050, MA at 40×).

### *In vitro* cell culture

hMSCs (passage #3–6) were cultured in complete media composed of Alpha Minimum Essential medium (*α*-MEM) (Gibco, Grand Island, NY) supplemented with fetal bovine serum (FBS) (16%, v/v) (Atlanta Biologicals, Lawrenceville, GA), L-glutamine (1% v/v) (Invitrogen, Carlsbad, CA) and penicillin:streptomycin (1% v/v) (Invitrogen, Carlsbad, CA). Cells were incubated under standard cell culture conditions (37 °C, 5% CO2 and 95% relative humidity). For chondrogenesis, 100 nM dexamethasone, 40 μg/mL proline, 100 μg/mL sodium pyruvate, 50 mg/mL L-ascorbic acid 2-phosphate and 1% ITS were added to the above complete media. Media were replaced every other day.

### Determination of hMSC proliferation under LIPUS and MB treatment

Cells were seeded at a density of 15 × 10^3^ per well in 24-well plates overnight before LIPUS and MB experiments to permit cell attachment to the plates. On the next day, the media were replaced to remove non-adherent cells. After LIPUS and MB treatments for predetermined periods, samples were rinsed using phosphate buffer saline (PBS) and the cells were lifted with 0.25% trypsin-EDTA solution. Cell proliferation was quantified via CellTiter 96 Aqueous Solution Cell Proliferation assay (MTS) (Promega, Madison, WI) and analyzed using a spectrophotometer at 490 nm (Thermo, USA)^81^.

### Determination of chondrogenic differentiation under LIPUS and MB treatment

All scaffolds for differentiation studies were seeded at a density of 1 × 10^5^ cells per scaffold. Prior to cell seeding, the samples were sterilized via UV exposure and then rinsed with PBS three times. Subsequently, the sterilized samples were pre-soaked in culture media for 24 hours before cell seeding. After predetermined time periods of LIPUS and MB treatments (the same as described above), these samples were collected after 1, 2 and 3 weeks to evaluate hMSC chondrogenesis. Media were removed from the samples and the latter rinsed with PBS. The collected samples were freeze dried in a lyophilizer and treated in a Papain digestion solution for 18 hours in a 60 °C water bath. Glycosaminoglycan (GAG) and collagen type II, which are two key components of cartilage matrix, were measured using standard assay kits following manufacturer’s instructions. Details of the process can be found in^81^,^82^.

### Ultrasound excitation

**Figure 8:**
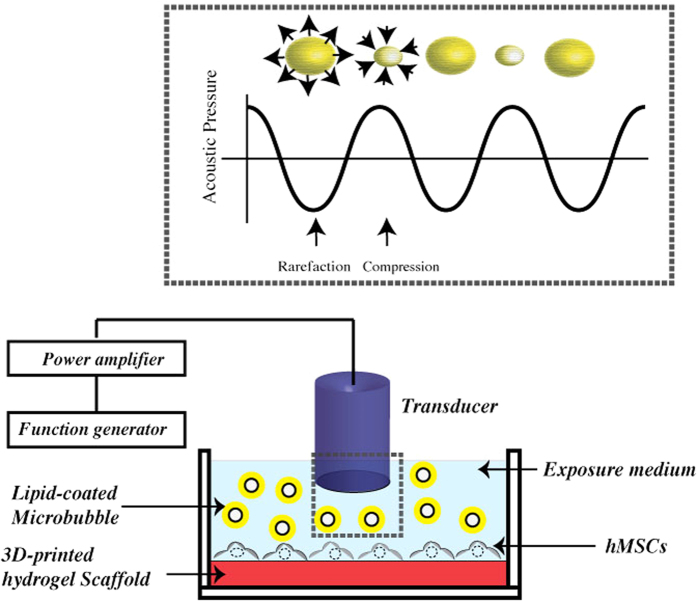
Schematic representation of LIPUS and microbubble exposure setup.

The schematic representation of ultrasound exposure setup is shown in Fig. 8. Briefly, the ultrasound pulse was produced by a programmable function generator (33250 A, Agilent, Palo Alto, CA, USA), amplified by a broadband 55 dB laboratory RF power amplifier (model A-150, ENI, Rochester, NY, USA) and then emitted from a single element unfocused immersion transducer. The outside diameter of the transducers was 16 mm. For frequency-dependent studies, we used unfocused immersion transducers (Olympus NDT, Waltham, MA) with central frequencies of 2.25 MHz (−6 dB: 1.48–2.90 MHz) and 3.5 MHz (−6 dB: 2.5–4.99 MHz). All the transducers were sterilized with 75% ethanol and kept under ultraviolet light overnight before the experiments.

To conduct this study, the transducer head was placed vertically on the top of the culture plate until it touched the media. In this configuration, the working distance of approximately 12 mm from the cell culture surface was fixed and kept constant throughout all experiments. Since the reflection coefficient of air-polystyrene interface is much higher than polystyrene-water interface, the 24-well plates were placed in a circular (5.5 inches in diameter) aluminum container filled with water.

Prior to ultrasound exposure the cells were washed two times with PBS. Ultrasound was applied to the cells after adding 3 mL of medium to each well. For cells being exposed to ultrasound and MBs, a mixture of culture media and MBs were first prepared separately and then pipetted into each well. To determine the optimal concentration of MBs as well as ultrasonic parameters we perform experiments varying intensity (10–300 mW/cm^2^), duty cycle (0.02–80%), frequency (1.5–3.5 MHz) and excitation duration (1–5 min) in the presence of MBs. Note that in all the experiments the pulse length is 200 μs. The duty cycle is the ratio of the actual pulse length to the pulse repetition period (PRP). The PRP is the inverse of the pulse repetition frequency (PRF). Control groups underwent the same submersion and withdrawal of transducers without any MB and with ultrasound power turned off.

### Statistical analysis

All proliferation studies were run in triplicate and repeated three times (n = 9). For the chondrogenic differentiation study, 5 replicates were performed (n = 5). Data are presented as mean ± standard error of the mean (StdEM) and analyzed by the Student’s t-test. A p < 0.05 was considered as statistically significant.

## Additional Information

**How to cite this article**: Aliabouzar, M. *et al*. Lipid Coated Microbubbles and Low Intensity Pulsed Ultrasound Enhance Chondrogenesis of Human Mesenchymal Stem Cells in 3D Printed Scaffolds. *Sci. Rep.* **6**, 37728; doi: 10.1038/srep37728 (2016).

**Publisher’s note:** Springer Nature remains neutral with regard to jurisdictional claims in published maps and institutional affiliations.
